# Effect of renin angiotensin blockers on angiotensin converting enzyme 2 level in cardiovascular patients

**DOI:** 10.1186/s40360-023-00667-w

**Published:** 2023-04-14

**Authors:** Sarah Farid fahmy, Marwa Omar El Derany, Hazem Khorshid, Ayman Saleh, Ebtehal El-Demerdash

**Affiliations:** 1 Department of Clinical Pharmacy, Faculty of Pharmacy, Ain Shams University, Cairo, Egypt; 2 Department of Biochemistry, Faculty of Pharmacy, Ain Shams University, Cairo, Egypt; 3 Department of Cardiology, Faculty of Medicine, Ain Shams University, Cairo, Egypt; 4 Department of Pharmacology and Toxicology, Faculty of Pharmacy, Ain Shams University, Cairo, 11566 Egypt; 5 Preclinical and Translational Research Center, Faculty of Pharmacy, Ain Shams University, Cairo, Egypt

**Keywords:** ACE2, ACEIs, ARBs, RAAS blockers

## Abstract

**Background:**

Renin–angiotensin–aldosterone system (RAAS) is hypothesized to be in the center of COVID pathophysiology as the angiotensin converting enzyme 2 (ACE2) represents the main entrance of the virus, thus there is a need to address the effect of chronic use of RAAS blockers, as in case of treatment of cardiovascular diseases, on the expression of ACE2. Accordingly, this study aimed to clarify the effect of ACE inhibitors (ACEIs) and angiotensin-receptor blockers (ARBs) on ACE2 and to assess the correlation between ACE2 and several anthropometric and clinic-pathological factors.

**Methods:**

A total of 40 healthy controls and 60 Egyptian patients suffering from chronic cardiovascular diseases were enrolled in this study. Patients were divided into 40 patients treated with ACEIs and 20 patients treated with ARBs. Serum ACE2 levels were assessed by ELISA.

**Results:**

Assessment of serum ACE2 level in different groups showed a significant difference between ACEIs and healthy groups and ACEIs and ARBs group, while there was no difference between ARBs and healthy. Multivariate analysis using ACE2 level as constant and age, female sex, ACEIs use and myocardial infarction (MI) showed that there was a significant effect of female sex and ACEIs use on ACE2 level with no effect of age, MI and diabetes.

**Conclusion:**

ACE2 levels varied between ACEIs and ARBs. It tends to be lower in ACEIs group and there is a strong positive association between ACE2 level and the female sex. This needs to be considered in Future studies to further understand the relationship between gender, sex hormones and ACE2 level.

**Trial registration:**

Retrospectively registered ClinicalTrials.gov ID: NCT05418361 (June 2022).

**Graphical abstract:**

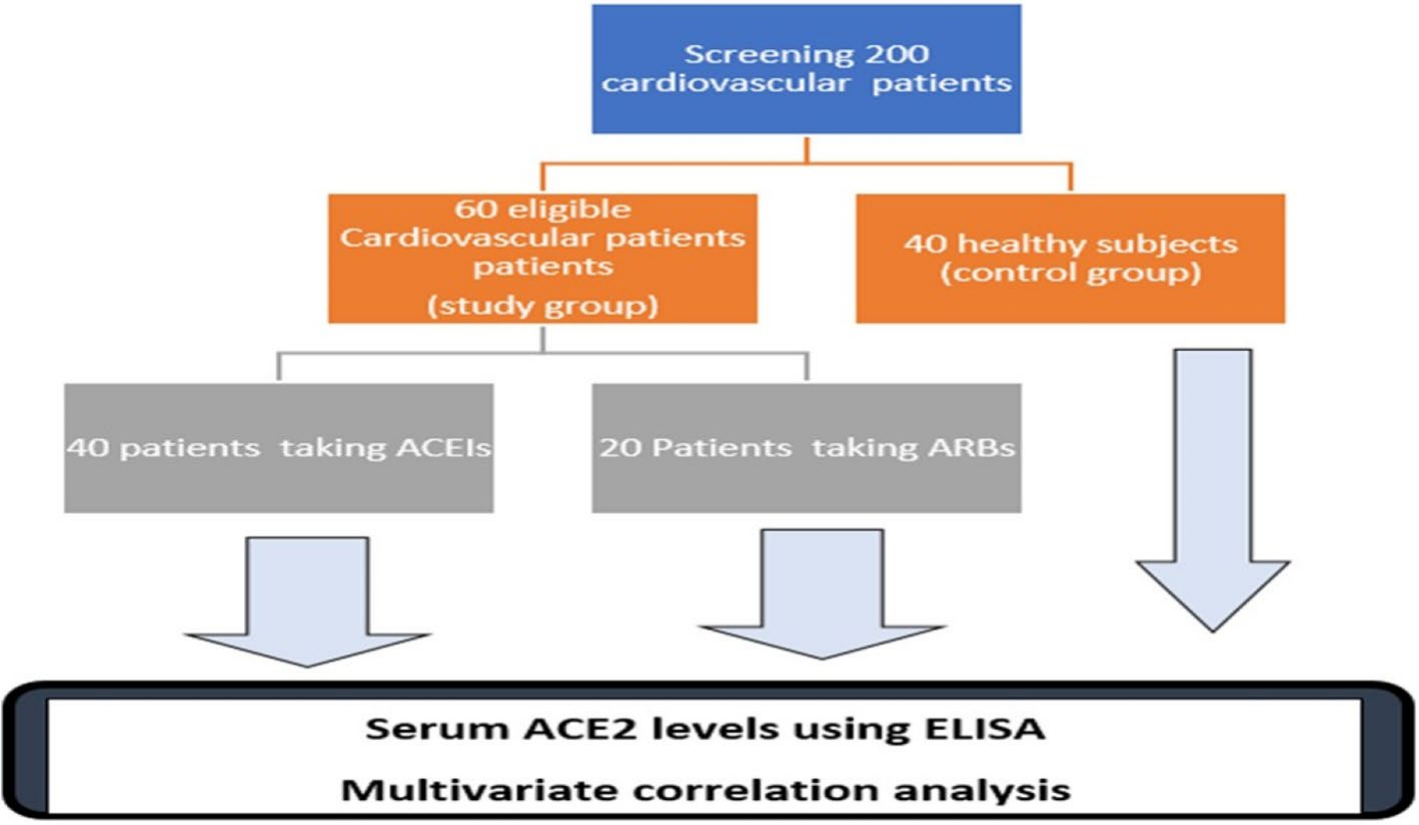

## Introduction

Blockage of renin–angiotensin–aldosterone system (RAAS) by either angiotensin-converting enzyme inhibitors (ACEIs) or angiotensin-receptor blockers (ARBs) is considered the first-choice of drugs for treatment of hypertension, heart failure and chronic kidney diseases [1]. In RAAS system, there are two pathways. The first classic one involves the cleavage of angiotensin I (Ang I) by dipeptidyl-carboxypeptidase angiotensin converting enzyme (ACE) to produce angiotensin II (Ang II) that acts on Ang receptors type 1 and 2 (AT1R and AT2R) and induces vasopressor effects. The other one is a counter-regulatory pathway which involves a mono-carboxypeptidase ACE2, which converts Ang II to angiotensin 1–7 (Ang 1–7), that binds to the Mas receptor (MasR), and counterbalance the classical Ang II–AT1R axis [2]. As Ang II is the vasopressor, both of ACEIs and ARBs are synthesized to counter the effect of Ang II and induce antihypertensive effect. However, the interplay between RAAS blockers and ACE2 hasn’t been fully elucidated [3].

Now, it is known that the entry of SARS-CoV into cells is mediated by the interaction between spike glycoprotein and the ACE2 to cause COVID-19. However, ACE2 is not only the entry receptor of the virus but also it protects from lung injury. Therefore, SARS-CoV became highly lethal because the virus deregulates a lung protective path [4]. One might consider that increased expression of transmembrane ACE2, could be interpreted as harmful, due to the possibility of favoring viral entrance. But, in view of the proposed pathophysiology, the association between SARS-CoV-2 and the bioavailability of ACE2 is rather paradoxical: either (1) the patient has a small ACE2 reservoir and suffers the consequences of the exacerbated pro-inflammatory classical Ang II–AT1R axis or (2) the individual infected has enough ACE2 to resist its depletion and to activate the alternative Ang 1–7-MasR axis, despite facilitating viral entrance. So, the conception of RAS imbalance pivotal role in COVID-19, has turned researchers to reconsider RAS blockers as potential therapies for the disease [5]. However, the effect of chronic use of RAAS blockers, as in case of treatment of cardiovascular diseases, on the expression of ACE2 is still not well characterized.

To date, conflicting evidences were reported linking the use of RAAS blockers and the susceptibility to the virus. However, others showed that treatment with an RAAS blockers may downregulate the expression of ACE2 but have no significant effect on its activity [6]. Furthermore, SARS-CoV-2 infection has largely been influenced by multiorgan involvement inducing multiple comorbidities due to the wide distribution of ACE2 thought out the whole body [7]. In addition, hypertension, cardiovascular disease, and chronic kidney disease are supposed to be potential but unconfirmed risk factors for COVID-19 in adults and children [8]. However, the expression of ACE2 may be lower in patients with hypertension than in people with normal blood pressure [6]. Concluding more conflicting evidences for the association of COVID-19 with RAAS inhibitors.

Given the RAAS importance to cardiovascular and kidney physiology, and the association between chronic diseases and COVID-19, could shed light on potential relationships between ACE inhibitor and ARB use and COVID-19 [9], this study aimed to clarify the effect of RAAS blockers on ACE2 and to assess the association between the chronic use of either ACEIs or ARBs on ACE2 level in clinical samples from patients with chronic cardiovascular diseases compared to healthy individuals. In addition, we studied the correlation between ACE2 and several clinical factors including age, body mass index, sex and diseases such as diabetes, Myocardial infarction, Hypertension and heart failure to explore the possible association between ACE2 level upon treatment with ACEIs or ARBs and these factors that may play role in COVID severity.

## Subjects and methods

### Study population and anthropometric measurements

This prospective case control study included a total of 100 subjects (57 men and 43 women) over a period of 8 months (from June 2021 to February 2022) recruited from Cardiology Department Rehabilitation clinic, Ain Shams University Hospital (ASUH), Cairo, Egypt. A total of 200 patients were screened according to following inclusion criteria:

1. Patients age > 18 years
2. Taking ACE II inhibitors or angiotensin II blockers (according to maximum tolerated dose specified by the American College of Cardiology guidelines) for more than 6 months. The maximum tolerated dose depends on disease state (heart failure or hypertension) and Drug type (ACEI or ARBs)
3. History of essential hypertension or heart failure.

Only Sixty subjects were being diagnosed with chronic cardiovascular diseases and being treated with either ACEI or ARBs were included in study. All patients underwent a full physical examination, heart rate measurement as well as left ventricular fraction (EF) by a cardiologist. Exclusion criteria include patients who suffer from secondary hypertension, including idiopathic hyperaldosteronism, renal artery stenosis. The exclusion criteria include also patient reported a history of severe liver disease or mental illness and a history of smoking, alcohol or any type of drug abuse.

In addition, 40 age matched healthy control subjects were enrolled in this study. A detailed interview was done by physician and clinical pharmacist about any medical and medication history. Full physical examination and heart rate measurement was done by cardiologist. Laboratory measurement of CBC, liver and kidney function tests were done for all subjects. These subjects were not suffering from any diseases and were not receiving any medications. They were family members of the patients with same lifestyle and environmental conditions. All subjects were informed about the study investigations, study protocol, potential risks, purpose and benefits of the study and a written consent was signed by all enrolled subjects. This study conforms with the principles outlined in the Declaration of Helsinki and was approved by the Ethical Committee of Faculty of Medicine (FWA 000,017,585) Ain Shams University, Cairo, Egypt.

### Blood sampling

Venous blood samples (5 ml) were collected on plain vacutainer tubes and left to clot for serum preparation. Serum were prepared by centrifugation at 4000 rpm for 10 min. The obtained serum samples were aliquoted and kept at -80 °C for subsequent use.

### Laboratory analyses

Serum human ACE2 levels were determined by commercially available ELISA kit (MyBioSource, San Diego, CA, USA) using Hyprep Automated ELISA system (Hyperion Inc, Miami, FL) according to the manufacturer’s instructions. The Sensitivity of human ACE2 is 1 pg/ml. Intra-assay CV is < 9% and nter-assay CV is < 10%. Routine Laboratory measurements of CBC, Fasting Blood glucose level, HBA1c, liver and kidney function tests were done in hospital labs.

### Sample size calculation

Sample size was calculated using NCSS PASS 11.0 and based on a study carried by Uri et al., 2016 [10]. Sample size of 50 participants (25 in Group1 of cardiovascular patients ACEI, 25 patients taking ARBs) and 25 healthy volunteers achieve 100% power to detect a difference of 19.1 between null hypothesis and alternative hypothesis and significance of alpha 0.05 using two sided t test.

### Statistical analysis

Statistical analysis was done using IBM SPSS® Statistics version 26 (IBM® Corp., Armonk, NY, USA). Numerical data were expressed as mean and standard deviation or median and range as appropriate. Qualitative data were expressed as frequency and percentage. Pearson’s Chi-square test or Fisher’s exact test was used to examine the relation between qualitative variables. For not normally distributed quantitative data, comparison between two groups was done using Mann–Whitney test (non-parametric t-test). Comparison between 3 groups was done using either analysis of variance (ANOVA) for normally distributed quantitative variables or Kruskal–Wallis test (non-parametric ANOVA) then post-Hoc test" was used for pair-wise comparison based on Kruskal–Wallis distribution. Spearman-rho method was used to test correlation between numerical variables. Multivariate analysis was done using multiple linear regression for the significant factors on univariate analysis. All tests were two-tailed. A *p*-value < 0.05 was considered significant.

## Results

Clinical characteristics of the study groups are shown in Table 1. The healthy group was characterized by normal heart performance and function, without any signs or symptoms of HF or any cardiovascular disorder, and were not treated with any pharmaceutics. The patient groups were chosen to represent the major steps in the cardiovascular continuum. Among the cardiovascular comorbidities in the HF group hypertension was the most common. There was non-significant difference between groups regarding age. While there was a significant difference regarding sex with females’ predominance (Table 1).

**Table 1:** Baseline characteristics

Groups		ACEIs	ARBs	Healthy	test value	*P*-value
**Parameters**						
**Demographics**						
**Age**	**Mean ± SD**	53.5 ± 9.6	54.5 ± 12.2	53.18 ± 8.15	0.106^b^	0.899
**BMI**		28.4 ± 5.2	27.9 ± 4	28.8 ± 5.8	0.184^b^	0.832
**Sex M**	**N (%)**	35 (87.5%)	13 (65%)	9 (22.5%)	35.129^a^	** < 0.001**
**Sex F**		5 (12.5%)	7 (35%)	31 (77.5%)		
**Comorbidities**						
**DM**	**No**	26 (65%)	13 (65%)		0^a^	1
	**Yes**	14 (35%)	7 (35%)			
**MI**	**No**	18 (45%)	20 (100%)		17.368^a^	** < 0.001**
	**Yes**	22 (55%)	0 (0%)			
**HTN**	**No**	21 (52.5%)	11 (55%)		0.033^a^	0.855
	**Yes**	19 (47.5%)	9 (45%)			
**EF**	**Normal**	20 (50%)	7 (35%)		4.108^a^	0.128
	**Medium**	12 (30%)	4 (20%)			
	**Reduced**	8 (20%)	9 (45%)			
**Drugs**						
**beta blockers**	**No**	24 (60%)	15 (75%)		1.319^a^	0.251
	**Yes**	16 (40%)	5 (25%)			
**Diuretics**	**No**	35 (87.5%)	19 (95%)			0.653**
	**Yes**	5 (12.5%)	1 (5%)			
**calcium blockers**	**No**	38 (95%)	19 (95%)			small number
	**Yes**	2 (5%)	1 (5%)			

*ACEIs* angiotensin converting enzyme inhibitors, *ARBs* angiotensin receptor blockers, *BMI* body mass index, *DM* diabetes mellitus, *HTN* hypertension, *EF* Ejection fraction

^a^chi square test,**Fischer exact, ^b^ANOVA test

Assessment of ACE2 level in different groups showed a significant difference between ACEIs and healthy groups (*p* = 0.006) and ACEIs and ARBs group (*p* = 0.001) using post hoc analysis. While there was no difference between ARBs and healthy controls (*p* = 0.716) (Table 2).

**Table 2:** ACE2 level in ACEI, ARBs and healthy groups

ACE2 level	ACEIs (*n* = 40)	ARBs (*n* = 20)	healthy (*n* = 40)	*P*–value
Mean ± SD (pg/ml)	**30.78 ± 20.46**	**62.11 ± 55.79**	**69.41 ± 96.25**	< 0.001*
Median	**25.17**	**49.57**	**29.27**	
(Max—Min) (pg/ml)	**(17.47—109.35)**	**(19.79—236.25)**	**(18.88—560.93)**	
Post hoc *P*-value between ACEIs and healthy	**0.006**			
Post hoc *P*-value between ACEIs and ARBs		0.001*		
Post hoc *P*-value between ARBs and Healthy			**0.716**	

*ACE2* Angiotensin converting enzyme 2, *ACEIs* angiotensin converting enzyme inhibitors, *ARBs* angiotensin receptor blockers

^*^P-value is statistically significant using Kruskal Wallis test

Concerning the age, there were no differences in ACE2 level between patients in ACEIs or ARBs groups less than 55 years or older age > 55 years of age (*p* = 0.099).

**Fig. 1:**
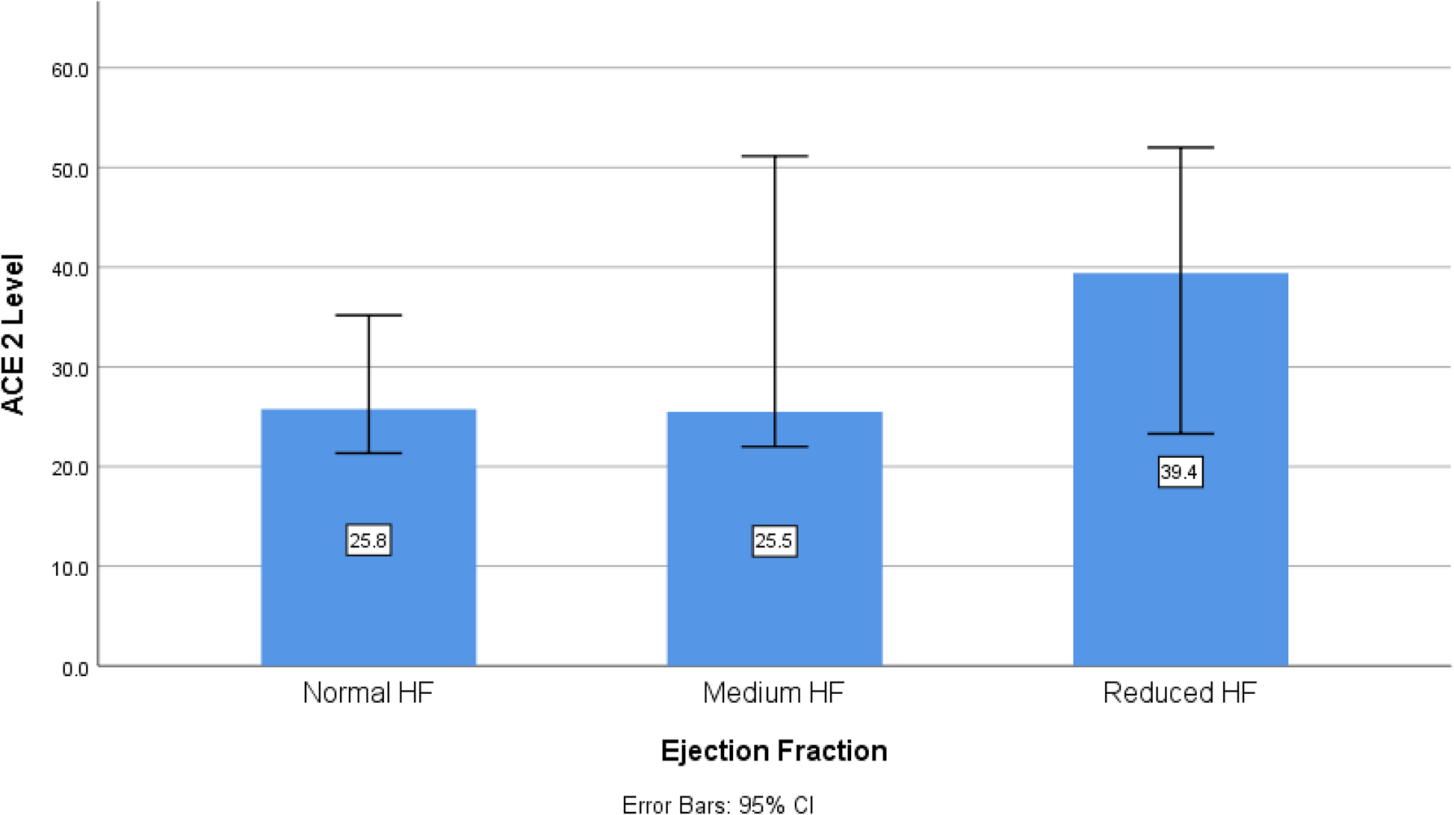
ACE2 level in Heart failure patients with normal, medium and reduced ejection fraction using kruskal wallis test

Similarly, there was no difference among heart failure patients with preserved, medium or reduced ejection fraction (*P* = 0.723). However, there was weak positive correlation between age and ACE2 level (R = 0.258, *P* = 0.047). On the other hand, there was no correlation between ACE2 level and BMI (R = -0.167, *P* = 0.736) or ejection fraction (R = -0.167, *P* = 0.203) (Fig. 1).

Table 3 showed the effect of several factors on ACE2 levels. Only the female sex has significant higher levels of ACE2 than male sex (*p* < 0.001). There was no significant difference regarding ACE2 levels between diabetic and non-diabetic patients (*p* = 0.687) or between hypertensive and non- hypertensive patients (*p* = 0.121). Patients with myocardial infarction showed significant lower ACE2 levels compared to the other patients (*p* = 0.002). Comparing ACE2 levels in patients on beta blockers or diuretics versus patients not taking them, there was no statistical difference in ACE2 levels. Regarding the effect of Ejection fraction on ACE2 levels, there was no difference between ACE2 levels in patients with either normal, medium or reduced EF (*p* = 0.626) (Table 3).

**Table 3:** Effect of Different parameters on ACE2 level

	Parameters		Median (min–max)	test value	*P*-value
ACE2 level (pg/ml)	**Sex (males)**		25.23 (17.47—57.12)	51	** < 0.001**
	**Sex (females)**		56.68 (27.97—236.25)		
	**DM**	**No**	25.88 (18.5—236.25)	383.5	0.687
		**Yes**	26.92 (17.47—109.35)		
	**MI**	**No**	35.59 (19.4—236.25)	214.5	**0.002**
		**Yes**	24.91 (17.47—55.18)		
	**HTN**	**No**	25.36 (17.47—109.2)	343.25	0.121
		**Yes**	26.99 (17.85—236.25)		
	**beta blockers**	**No**	27.05 (17.85—236.25)	364.5	0.486
		**Yes**	25.49 (17.47—196.02)		
	**diuretics**	**No**	26.08 (17.85—236.25)	142.5	0.639
		**Yes**	29.28 (17.47—109.35)		
	**EF**	**Normal**	25.75 (18.5—236.25)	0.936^a^	0.626
		**Medium**	25.49 (17.85—109.35)		
		**Reduced**	39.4 (17.47—56.12)		

*ACE2* Angiotensin converting enzyme 2, *ACEIs* angiotensin converting enzyme inhibitors, *ARBs* angiotensin receptor blockers, *BMI* body mass index, *DM* diabetes mellitus, *HTN* hypertension, *EF* Ejection fraction

^a^Done by Kruskal Wallis test, others test values using Mann Whitney test

Due to the significant effect of sex on ACE2 level (*p* < 0.001) as well as myocardial infarction (*p* = 0.002), multivariate analysis was done using ACE2 level as constant and age, female sex, ACEIs use and MI as variables. There was significant effect of female sex and ACEIs use on ACE2 level with no effect for age and MI (Table 4).

**Table 4:** Multi linear regression analysis

Model	T	*p*-value	95.0% Confidence Interval for B	
			Lower Bound	Upper Bound
(ACE2level Constant)	-2.37	0.021	-126.121	-10.551
Age	0.719	0.475	-0.482	1.022
MI	0.401	0.69	-16.005	24.006
ACEI use	2.146	**0.036**	1.383	40.502
female gender	5.217	** < 0.001**	33.693	75.721

## Discussion

ACE2 is one of the main enzymes of the RAAS, which plays central role in cardiovascular system regulating blood pressure and vascular tone [11]. ACE2 levels and its expression were used as prognostic markers for multiple organ functions especially heart and lungs [12]. Recently, ACE2 has gained attention from cardiologists with debates on the use of ACEIs and ARBs and their effect on people infected with SARS-CoV-2 [11]. The clinical signs and symptoms of COVID-19, a complicated illness, are consistent with those that would be anticipated in response to excessive Ag II exposure. This is due to the internalization process of ACE2 after binding to the viral S protein and consequently, preventing its counter-regulative effect mediated by the metabolism of angiotensin II to angiotensin (1–7) [13]. As Ang II is the vasopressor, both of ACEIs and ARBs are synthesized to counter the effect of Ang II and induce antihypertensive effect. However, the interplay between RAAS blockers and ACE2 hasn’t been fully elucidated [11].

In the present study, we investigated the serum ACE2 levels in established cardiovascular patients treated with either ACEIs or ARBS and compared them with healthy individuals. It was found that, there is a significant difference between ACEIs and healthy subjects and between ACEIs and ARBs. The patients on ACEIs has the lowest ACE2 levels amongst the three groups. In addition, there is no difference between ARBs and healthy subjects. In previous studies, ACEIs treatment was also independently associated with significantly lower ACE2 levels in CHANTI study [14]. This was in line with a previous animal study in which an IV infusion of lisinopril and losartan increased cardiac ACE2 mRNA and gene transcription with double increase in lisinopril group compared to losartan group. This postulated that the numbers of ACE2 receptors may be increased in patients with cardiovascular diseases taking ACEIs or ARBs [15]. Also, Furuhashi et al. showed that the urinary ACE2 level was increased in hypertensive patients treated with olmesartan but not with other ARBs (losartan, candesartan, valsartan, and telmisartan) or with the ACEI; enalapril, compared to untreated control patients [16].

However, in a large cohort of patients with established cardiovascular disease or cardiovascular risk factors but without heart failure, ACEIs and ARBs were not associated with higher serum levels of ACE2 [17]. Also, in an European heart failure study, it was found that the use of neither ACEIs nor ARBs was associated with higher ACE2 levels [18]. Further, patients with coronary artery disease did not influence angiotensin-(1–7) production when administered ACEIs by intravenous infusion [19]. In addition, a previous study of paroxysmal atrial fibrillation showed that there was no association between ACEIs or ARBs use with ACE2 level [20]. Pharmacologically, it is expected that while ACEIs may induce a decrease in ACE2 level, the ARBs will not because ACEIs act through inhibition of ACE enzyme that responsible for synthesis of Ag-II and as ACE2 is a counter regulatory pathway that responsible for changing Ag-II to Ag (1–7), it will not increase in case of inhibiting synthesis of Ag-II.

To further explore the possible association between ACE2 level upon treatment with ACEIs or ARBs and the clinical factors that may play role in COVID severity, we studied the correlation between ACE2 and several clinical factors including age, BMI, sex and diseases such as diabetes, MI, HTN and HF. It was found that there was no significant difference in the ACE2 level between patients aged less or greater than 55 years. Our results are in contrast to the study of Chirinos et al. (2020) who reported an association between lower ACE2 level and older age (above 50 years), male sex and diabetes [21], however, they did not detect any significant difference between the effect of ACEIs and ARBs on the ACE2 level. In other studies, circulating ACE2 levels demonstrated curvilinear association with age, with older individuals beyond the sixth decade age having lower levels [14]. Also, ACE2 nasal gene expression was found to be increased linearly from < 10 to 18–24 years but with less pronounced increase among those aged 25 years or older [22]. Accordingly, we cannot consider the age as predictor factor for the level of ACE2.

Regarding the sex factor, interestingly, our study showed that female sex could be a predictor for ACE2 level. This was in contrast to the European heart failure study [18] and Chirinios et al. study [21] that showed a sex hormone-dependent greater ACE2 expression in men compared to women. However, Zimmermann et al., (2021) study failed also to find that male patients had lower ACE2 levels than females [17]. Further, in an Italian study, the ACE2 activity was not correlated with gender difference among patients with COVID 19 [23]. These results may need further investigation as there were difference in distribution between males and females in both groups of our study.

It was believed that the increase in body mass index may affect ACE2 levels. However, in the present study, we found no association between BMI and ACE2 levels. This finding is in accordance with CHANTI study [14], while other studies showed positive association between ACE2 level and BMI [17]. Regarding the effect of diabetes on ACE2 level, there was no significant difference in the ACE2 level among diabetic and non-diabetic patients and this finding is in agreement with previous studies [10, 14], while Chirinios et al. study showed that diabetes had significant effect on ACE2 level compared to non-diabetic [21].

Since ACE2 has been implicated in the pathophysiological mechanisms of various cardiovascular diseases, further correlation was tested between ACE2 level and different cardiovascular diseases. In the present study, it was found that there was no significant difference between ACE2 levels in hypertensive and non- hypertensive patients. Patel et al. had also reported no association between circulating ACE2 activity and hypertension [24]. Similarly, we found no significant difference in ACE2 levels among heart failure patients with normal, medium and reduced ejection fraction. But this finding is in contrast to eprlmen study in which the ACE2 activity is elevated in HF patients with increased ejection fraction relative to healthy controls and correlates with worsening left ventricular ejection fraction [25].

In recent years, multiple studies have shown that the RAAS, especially ACE2, is involved in MI-induced myocardial remodeling [26]. In the early stage of MI, both ACE2 and ACE levels are remarkably increased in the heart, whereas in the late stage, ACE2 expression declines and is accompanied by HF, indicating the role of ACE2 against the RAAS [26]. In the present study, we found a significant difference between ACE2 levels in MI versus non MI patients. Similarly, clinical research has found that the serum ACE2 level of MI patients is significantly higher than that of healthy individuals and results in a negative prognosis. Further, the results indicate that the serum level of ACE2 may be a candidate for identifying the degree of myocardial injury [27].

Finally, at the present time, it is well known that, one of the factors that is responsible for exacerbation of pro-inflammatory cytokines in COVID-19 is the activation of local RAS in the lung leading to generation of Ag-II by ACE that acts on Ang-II receptors. On the other hand, the counter-regulatory pathway involves the ACE2, which converts Ang II to angiotensin 1–7, that binds to the Mas receptor [3, 11]. Thus, one of the important findings of the present study is to clarify the effect of ACEIs or ARBs upon chronic administration, on ACE2 levels and this may help in evaluating the benefit from using these drugs in case of COVID-19. Several retrospective studies reported an association between the use of ACEIs and ARBs and a delay in the progression of pulmonary complications with improved pneumonia-related outcomes [28, 29]. In general, the potential anti-inflammatory and antifibrotic effects of different ACEIs and ARBs have been approved in other organs, in particular the liver [30]. In addition, several meta-analysis studies support the protective effect of RAAS inhibitors against clinical symptoms in COVID-19 patients. However, the collective data of the present study confirmed that there may be a differential effect between ACEI and ARBs on ACE2 that needs to be further studied in case of COVID-19.

## Conclusion

ACE2 levels varied between ACEIs and ARBs. It tends to be lower in ACEIs group and there is a strong positive association between ACE2 level and the female sex. This needs to be considered in future studies to further understand the relationship between gender, sex hormones and ACE2 level. Also more studies are needed to examine the implications of these differences in cohorts with ACE2 levels and COVID-19 outcomes. More detailed characterization of the different components of RAAS signaling is needed with a focus on absolute quantitative assessment in larger cohort toward establishing normative values, and assessment of their longitudinal changes.

## Data Availability

The datasets used and/or analyzed during the current study are available from the corresponding author on reasonable request.
